# Subtle Distinctions: How Attentional Templates Influence EEG Parameters of Cognitive Control in a Spatial Cuing Paradigm

**DOI:** 10.3389/fnhum.2018.00113

**Published:** 2018-03-23

**Authors:** Christine Mertes, Daniel Schneider

**Affiliations:** Leibniz Research Centre for Working Environment and Human Factors, TU Dortmund, Dortmund, Germany

**Keywords:** attentional templates, top-down control, visual search, N2pc, P_D_

## Abstract

Using event-related potentials (ERPs) of the electroencephalogram, we investigated how cognitive control is altered by the scope of an attentional template currently activated in visual working memory. Participants performed a spatial cuing task where an irrelevant color singleton cue was presented prior to a target array. Blockwise, the target was either a red circle or a gray square and had to be searched within homogenous (gray circles) or heterogeneous non-targets (differently colored circles or various shapes). Thereby we aimed to trigger the adoption of different attentional templates: a broader singleton or a narrower, more specific feature template. ERP markers of attentional selection and inhibitory control showed that the amount of cognitive control was overall enhanced when participants searched on the basis of a feature-specific template: the analysis revealed reduced selection (N2pc, frontal P2) and pronounced inhibition (negative shift of frontal N2) of the irrelevant color cue when participants searched for a feature target. On behavioral level attentional capture was most pronounced in the color condition with no differentiation between the task-induced scopes of the attentional template.

## Introduction

Imagine you are walking down a busy city street—information flows in from all sides. This overwhelming quantity of sensory input is a great deal too much for our attentional system to handle simultaneously. Attention acts as a filter by keeping out irrelevant information, thereby enabling the selection of those environmental inputs that foster goal-directed behavior (Broadbent, 1958; Treisman, 1969). It is assumed that whenever we search for specific information in our visual surrounding, attention is guided by so-called attentional templates (Duncan and Humphreys, 1989) or top-down control sets (Folk et al., 1992): these mental representations are activated in visual working memory and influence processing by biasing neural competition in favor of the currently relevant objects (Bundesen, 1990; Duncan and Humphreys, 1992; Desimone and Duncan, 1995; Chelazzi et al., 1998). In the present study, we aimed to investigate how the scope of such an attentional template (i.e., the amount of top-down control) can vary during visual search.

It is widely accepted that the attentional system is quite flexible with regard to the stimulus properties it can be set to respond to Bacon and Egeth (1994), Folk and Anderson (2010) and Irons et al. (2012). Attentional templates can be either set to support the selection of any salient information in the environment or to guide attention toward information that possesses specific features (Bacon and Egeth, 1994). In this regard, researchers have addressed the question which circumstances exactly determine the implementation of a particular scope of an attentional template. At which point do we switch from a broader set to a more fine-tuned template and vice versa? Given that the general aim in attentional processing is to reduce load in a capacity limited system, the need for extended cognitive control should be kept to a minimum. It was proposed that broader templates are implemented as a kind of default mode as they place less cognitive demands on the attentional system (Bacon and Egeth, 1994). In contrast, a more fine-tuned attentional template implies a greater amount of cognitive control (Bacon and Egeth, 1994; Barras and Kerzel, 2016) and should only come into play when the sought-after object can no longer be detected on the basis of a broader template.

One opportunity to investigate the scope of an attentional template is to look how irrelevant information is handled during search for a predefined target. Sensory inputs possessing target-related features are preferentially selected regardless of whether they are relevant or irrelevant (Folk et al., 1992). According to this notion, we know that irrelevant information capturing attention is part of the currently activated attentional template. This should allow for a conclusion to be drawn about the scope of this template.

Well-suited experimental designs to consider the processing of irrelevant information are so-called spatial cuing paradigms (Folk et al., 1992, 1994; Eimer and Kiss, 2008). In a spatial cuing task, participants are instructed to search for a specific target in a search array which is preceded by a display containing an irrelevant, spatially non-predictive item. Depending on whether the irrelevant cue matches the target defining properties (contingent condition) or not (non-contingent condition), it will automatically summon attention to its location. These attentional capture effects typically become apparent through modifications in the response to the search target: in the contingent condition, response times (RTs) were found to be faster for trials with cue and target subsequently presented at the same location compared to those trials where they were presented at different locations. This offset in RTs between same and different location trials is known as the cuing effect (Folk et al., 1992, 1994; Eimer and Kiss, 2008) and proves that attention was already allocated to the location of the irrelevant, but task-set contingent cue, thereby facilitating the processing of the target subsequently presented at the same location.

However, findings from behavioral data alone leave room for speculations on the underlying attentional mechanisms during attentional orienting and accordingly, the amount of cognitive control that was engaged to select the target. By recording the electroencephalogram (EEG) during a spatial cuing paradigm, researchers found evidence for markers of attentional selection and inhibition in response to the irrelevant cue. A contralateral negativity starting approximately 180 ms after the onset of the cue array, namely an N2 posterior contralateral component (N2pc; Luck and Hillyard, 1994b), marks the selection of the irrelevant information. N2pc is especially pronounced when cue and target are contingent on the attentional template (Eimer and Kiss, 2008, 2010; Lien et al., 2008; Huang et al., 2016; Mertes et al., 2016, 2017; Schönhammer et al., 2016). Eimer and Kiss (2010) modified a spatial cuing paradigm to trigger the adoption of different attentional templates. Behavioral cuing effects and N2pc components were observed in response to irrelevant singleton cues when participants were allowed to adopt a broad template in order to find the target in the search array. In contrast, when participants were instructed to search for a target possessing a specific characteristic (in this case color), cuing effects together with N2pc components were solely triggered by the irrelevant cue that matched this particular feature. This indicated an increased amount of cognitive control during search.

Even if N2pc helps distinguishing the scope of an attentional template, the mere manifestation of this component cannot reveal if there are differences in the handling of irrelevant information between broader and more fine-tuned templates once attention was captured in a task-set contingent way. Thus, it might be useful to additionally consider markers of inhibitory control: a contralateral positivity (distractor positivity, P_D_) that arose in response to an irrelevant or distracting stimulus has been interpreted as an index of suppression (Hickey et al., 2009; Hilimire et al., 2012). By varying the stimulus onset asynchrony (SOA) between cue and target display in a spatial cuing paradigm, Mertes et al. (2016) were able to observe two independent contralateral positivities after attentional capture by the cue. A first positivity following N2pc was interpreted as mirroring an inhibitory process to neutralize attentional orienting to the irrelevant cue. In line with the functional meaning of P_D_, this first positivity was probably engaged to allow for a rapid disengagement from the cued location in order to enable an adequate processing of the upcoming target. A second positivity showed up during target array presentation and was assumed to index the suppression of spatial cue information that was erroneously transferred into working memory (see also Sawaki and Luck, 2013). Nevertheless, there exists some ambiguity regarding the inhibitory function of this late positivity. Long-standing evidence suggests that such a positivity is rather the product of a contralateral P1 enhancement that occurs whenever attention is oriented to the left or right prior to the onset of a bilateral stimulus array (Heinze et al., 1990; Luck et al., 1990; McDonald et al., 2005; Fukuda and Vogel, 2009; Störmer et al., 2009). This contralateral relative to ipsilateral amplification in the time range of P1 has been associated with attentional enhancement of the stimulus that appeared at the cued location. Recently, Livingstone et al. (2017) replicated a spatial cuing study that was conducted by Sawaki and Luck (2013) to uncover whether the positivity that occurred during target presentation was really a cue-elicited P_D_ or rather indexed attentional enhancement of the cued stimulus. Like Mertes et al. (2016), Livingstone et al. (2017) varied the SOA between cue and target array onset and could also show that the contralateral positivity was always linked to the onset of the target array. Based on their findings, the authors assumed that this target-tied P_D_ actually reflected a contralateral enhancement of P1 in response to the stimulus in the search array that occurred at the same location as the preceding irrelevant cue (Heinze et al., 1990; Luck et al., 1990; McDonald et al., 2005; Fukuda and Vogel, 2009; Störmer et al., 2009). Therefore, Livingstone et al. (2017) concluded that rather than indexing inhibition, this contralateral positivity (short CP) in fact reflects a boost in the perceptual processing of the cued stimulus in the search array. They argued that target processing benefited from this perceptual enhancement on same location trials whereas behavioral costs occurred on different location trials, thereby providing an explanation for the cuing effect on behavioral level.

**Figure 1:**
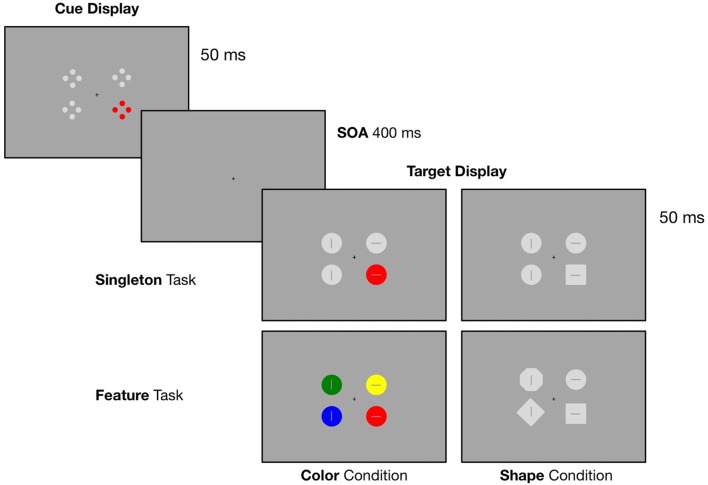
Illustration of the experimental design. Target displays were preceded by a cue display containing an irrelevant red singleton. In the singleton task the target was either a red circle (color condition) or a gray square (shape condition) presented within homogenous gray circles. In the feature task, participants had to respond to a red circle presented within differently colored circles (color condition) or a gray square within heterogeneous shapes (shape condition). Participants had to indicate the orientation of the line inside the target (horizontal vs. vertical) via left or right bottom press.

In the current study, we implemented a spatial cuing paradigm with four distinct blocks where we modified the target defining features as well as the context in which the target stimulus was presented. In all conditions, search displays were preceded by an irrelevant cue display containing one red (i.e., the irrelevant singleton) and three gray laterally presented stimuli (see Figure 1). In order to allow the adoption of a broader attentional template, we implemented a singleton task where the target was either defined as a red circle (singleton color condition) or a gray square (singleton shape condition) surrounded by gray circles. To provoke the adoption of a purely feature-specific attentional template, we added the feature task: in one block, participants had to respond to a red circle presented within differently colored circles (feature color condition) and in another block, they had to search for a square within heterogeneous shapes (feature shape condition).

Measuring the event-related potentials (ERPs) in response to the irrelevant cues was expected to reveal gradations in the amount of top-down control between the different conditions. In the singleton task, the target could be either selected by using the broad template of looking for the odd element in the search display or by activating a search template that incorporates only the specific target feature. If participants opt for a broader template, N2pc and P_D_ components in response to the color cue should occur in both, the color and the shape condition. Otherwise, if search proceeds on the basis of a fine-tuned template, cue-related attentional orienting and inhibitory effects should only occur in the color condition. In contrast, participants have to rely on a narrower attentional template in the feature task where targets do not possess a singleton status. Thus, top-down control should be overall enhanced in this case. Only in the color condition, the irrelevant cue meets the criteria of the attentional template and should trigger an N2pc component. Like in the study of Mertes et al. (2016), P_D_ should follow N2pc in order to compensate for attentional capture by the irrelevant cue. The following late contralateral positivity that might either reflect attentional enhancement at the cued location or inhibition of spatial cue information during target array presentation should be observable whenever the irrelevant cue matches the search template currently activated in working memory. Thus, this effect should also vary with the amount of top-down control applied during search: in all cases where the irrelevant cue triggers an N2pc, we should observe a more pronounced contralateral P1 to the stimulus in the target array that appeared at the same location than the preceding color cue.

Further insights into the variations of top-down control during search might be gained by considering markers of attentional orienting at frontal sites: attentional selection and in-depth processing of information is reflected by the anterior P2 component (Kenemans et al., 1993; Anllo-Vento and Hillyard, 1996; Verleger et al., 2014), whereas inhibitory control of irrelevant information (Falkenstein et al., 2002; Folstein and Van Petten, 2008; Eimer et al., 2009) or conflict processing (van Veen and Carter, 2002) is related to the frontal N2 component. Enhanced top-down control of attentional capture by the irrelevant cue should, therefore, be reflected in pronounced anterior N2 amplitudes and might also lead to modifications in the frontal P2 component.

At the behavioral level, spatial cuing effects should be observable in the contingent color condition of both the singleton and feature task. In the feature shape condition where the cue is not part of the activated template, cuing effects should be absent. For the singleton shape condition, we expect capture at the behavioral level to vary as a function of the amount of top-down control engaged during search. If participants use a broad attentional template and, thereby, apply low top-down control, cuing effects should be evident in this condition. The non-obligatory use of a feature-specific attentional template and, thereby, of higher top-down control would however be associated with the absence of cuing effects.

By focusing on the handling of irrelevant information during visual search we aimed to contribute to a better understanding of how cognitive control is adjusted to guarantee the achievement of behavioral goals.

## Materials and Methods

### Participants

Twenty-four participants (12 female, mean age = 24.04 years; SD = 2.68; range 19–29 years) took part in the experiment. None of them reported any neurological or psychiatric problem. All had normal or corrected to normal vision and color-blindness was excluded by means of the Ishihara color blindness test (Ishihara, 1991). Three participants were left-handed, the remaining ones were right-handed. Participants received course credits or a payment of 10 Euros per hour and provided informed written consent before the beginning of the experiment. The experiment was carried out in accordance with the Declaration of Helsinki. The local ethic committee of the Leibniz Research Centre for Working Environment and Human Factors endorsed the study.

### Stimuli and Procedure

In a dimly lit chamber, participants were seated in front of a 20″ CRT monitor with a refreshing rate of 100 Hz. The screen was set up with a distance of 145 cm. Presentation of the stimuli was controlled by a VSG 2/5 graphic accelerator (Cambridge Research System, Rochester, UK). Each trial started with the presentation of a cue array that was displayed for 50 ms. Followed by a SOA of 400 ms, the target array occurred and was presented for 50 ms. A small black fixation cross was constantly visible during the trial sequence (see Figure 1 for an illustration of the experimental design). The cue array was composed of four stimuli, two left and two right from fixation. Each stimulus was presented at a constant distance of 1.44° (of visual angle) from the central fixation cross. The array contained a red color singleton cue (CIE color coordinates = 0.566/0.376) together with three gray stimuli (CIE color coordinates = 0.287/0.312). The colored cue stimulus was randomly presented at one of the four lateral positions. Closely aligned to the cuing paradigm used by Eimer and Kiss (2008), each of the four cue stimuli consisted of four outlined dots (0.25° diameter) that were arranged in form of a diamond. Overall, there were four possible target array conditions: in all conditions the array included four laterally presented stimuli. All target stimuli were point symmetric and contained either a horizontal or vertical bar (length 0.375°, 0.0625° wide) in their center. Like the colored cue stimulus, the target stimuli were randomly presented at one of the four lateral positions. In the *singleton color condition*, the target array was composed of one red target circle presented together with three gray circles (0.75° diameter). A gray square (0.665° side length) presented among gray circles served as the target in the *singleton shape condition*. For the *feature color condition*, participants had to search for a red circle among three differently colored circles (either blue (CIE color coordinates = 0.168/0.131), green (CIE color coordinates = 0.292/0.574) or yellow (CIE color coordinates = 0.384/0.477)). In the *feature shape condition*, the target was a gray square presented among heterogeneous shapes (either a diamond, 0.665° side length, circle, or octagon, 0.3025° side length). Participants had to report the orientation of the line inside the target by key press with the index finger of the left or the right hand (e.g., line horizontal—right key press, line vertical—left key press), with the assignment of line orientation and response hand counterbalanced across participants. The responses were measured with force-sensitive keys and recorded along with the EEG.

Participants performed four consecutive experimental blocks (singleton color, singleton shape, feature color and feature shape condition), with each block including 320 trials. A 2-min break was made after every 160 trials. Therefore, each experimental session included eight sub-blocks. In order to reduce the possibility that participants adopt a feature search strategy in the singleton color condition, the experiment always started with the singleton task. The order of color vs. shape conditions was counterbalanced across participants. The stimuli in the cue and target arrays were presented with equal probability at one of the four lateral positions. Thus, the color singleton cues were spatially uninformative regarding the location of the target in the search array (25% validity).

Cue and target stimuli were presented on a dark gray background (10 cd/m^2^) and were matched for luminance (25 cd/m^2^). The luminance was calibrated with a ColorCAL MKII Colorimeter (Cambridge Research Systems). In order to adjust the luminance, values were measured on the monitor and then adopted in the VSG graphic accelerator.

### Data Analysis

#### Behavioral Data

Error rates and RTs were measured for the two target dimensions (color and shape) in the two tasks (singleton and feature). RTs were averaged across vertical cue-target location, horizontal cue-target location and diagonal cue-target location trials to an overall different cue-target location condition. For the same cue-target location condition only those trials were included where cue and target were subsequently presented within the same quadrant. Incorrect assignments of response side to the orientation of the line in the target were considered as discrimination errors. Missed responses (no response within 1000 ms following the onset of search array) were separately analyzed as omissions. Additionally, RTs shorter than 150 ms were categorized as fast guesses and also treated as errors. Analysis of variance (ANOVA) with the within-subjects factors *task* (singleton, feature), *target dimension* (color, shape) and *cue-target location* (same location, different location) were used to test for differences in error rates and RTs.

#### EEG Data

Sixty active Ag/AgCl electrodes (ActiCap; Brain Products, Gilching, Germany) according to the extended 10/20 System (Pivik et al., 1993) were used to record the EEG. The horizontal and vertical electrooculogram (EOG) were measured by affixing two additional electrode pairs at the outer canthi of each eye and above and below the left eye. EEG and EOG were sampled at 1000 Hz by a BrainAmp DC-amplifier with a low-pass filter of 250 Hz. Impedances were kept below 10 kΩ. Fpz served as ground electrode and reference was set to P9 during recording. The EEG analyses were conducted using MATLAB^©^ and the related packages EEGLAB (Delorme and Makeig, 2004) and ERPLAB (Lopez-Calderon and Luck, 2014) for EEG/ERP data analysis. The data were re-referenced offline to the average of the left (TP9) and right (TP10) mastoid electrode. Signals were filtered offline with a 0.5 Hz high-pass and a 20 Hz low-pass filter. To obtain the averaged ERP waveforms, epochs beginning 500 ms before and ending 1500 ms after cue display onset were chosen. The baseline was set to the 200 ms pre-stimulus period. Trials with incorrect responses as well as those with RTs shorter than 150 ms or longer than 1000 ms were excluded from all further analyses. An independent component analysis (ICA) was conducted to correct for eye-movement artifacts and discontinuities in the EEG data. We used ADJUST (Mognon et al., 2011) in order to automatically exclude ICs with artifacts.

Markers of attentional selection (N2pc), inhibition (P_D_) and late inhibition or perceptual enhancement (i.e., CP) were measured over the visual cortex at posterior electrodes PO7 and PO8. We computed the activation contra- and ipsilateral to the singleton cue in the first display. Waveforms were constructed by collapsing over the different trial types (same cue-target location, vertical cue-target location, horizontal cue-target location, diagonal cue-target location). Event-related lateralizations (ERLs) were calculated the same way the lateralized readiness potential is computed (Coles et al., 1988; Wascher and Wauschkuhn, 1996) by subtracting the ipsilateral from the contralateral signal. For statistical analyses, we measured the positive or negative area under the ERL difference wave over the time interval of interest for each component and averaged it across participants (Sawaki et al., 2012; Mertes et al., 2016). For N2pc the area below zero in the respective time range was calculated. Respectively, for positive waveforms we measured the area above zero. For negative area measurements, all positive values in the respective time range were zeroed and vice versa. This calculation resulted in an area value unit of μVs (microvolt seconds). Such an area measurement always produces values that are different from zero that could, however, be the product of random noise in the data. In order to prove the statistical significance of the measured area values, we calculated a distribution of areas that would be expected if there were only random lateralized activation in the data. This distribution was estimated by means of permutation tests where we randomly assigned the side of the target for each trial, computed the resulting negative or positive area value under the ERL for each participant and finally averaged across all participants. This procedure was repeated 1000 times with different randomizations. Significant difference from chance level was assumed if the measured area value in the original data was higher than 95% of values from the random distribution.

N2pc in response to the cue was measured as the area under the negative going ERL difference wave in the time interval from 150 ms to 300 ms. P_D_ was analyzed by calculating the area in the time window from 250 ms to 400 ms after the onset of the cue array. Finally, the positive area in the time window from 480 ms to 630 ms was measured to assess the CP (see Sawaki and Luck, 2013; Mertes et al., 2016, 2017). In order to elucidate whether the late positivity was produced by a contralateral P1 enhancement, we additionally plotted the activation contra- and ipsilateral to the target in the search array as a function of cue target location (see Figure 4). Therefore, we combined same with vertical cue-target location trials (i.e., same side condition). Horizontal and diagonal cue-target location trials formed the different side condition. As we observed a stronger contralateral compared to ipsilateral positivity in the time window of the cue-locked P1, we measured this asymmetry as the positive area in the interval from 80 ms to 140 ms. Separate ANOVAs for the within-subjects factors *task* (singleton, feature), and *target dimension* (color, shape) were conducted to test for differences in area values of the four components.

**Figure 2:**
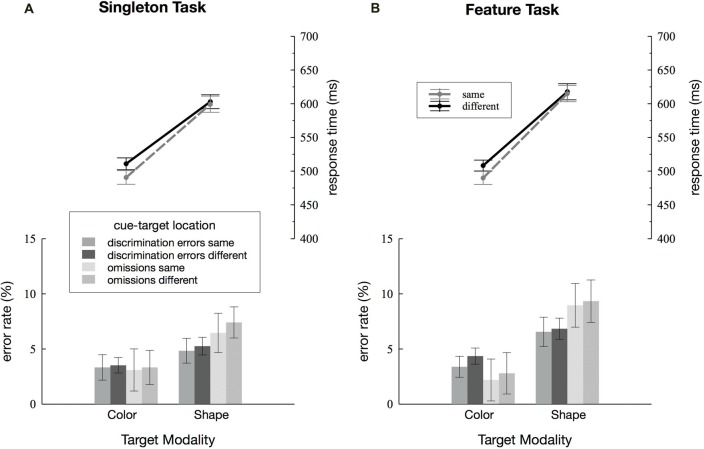
Behavioral results. Discrimination errors, omissions and response times (RTs) depending on cue-target location (same vs. different) and *target dimension* (color vs. shape) are separately depicted for the singleton **(A)** and feature task **(B)**. Error bars represent within-subject 95% confidence intervals (Morey, 2008).

**Figure 3:**
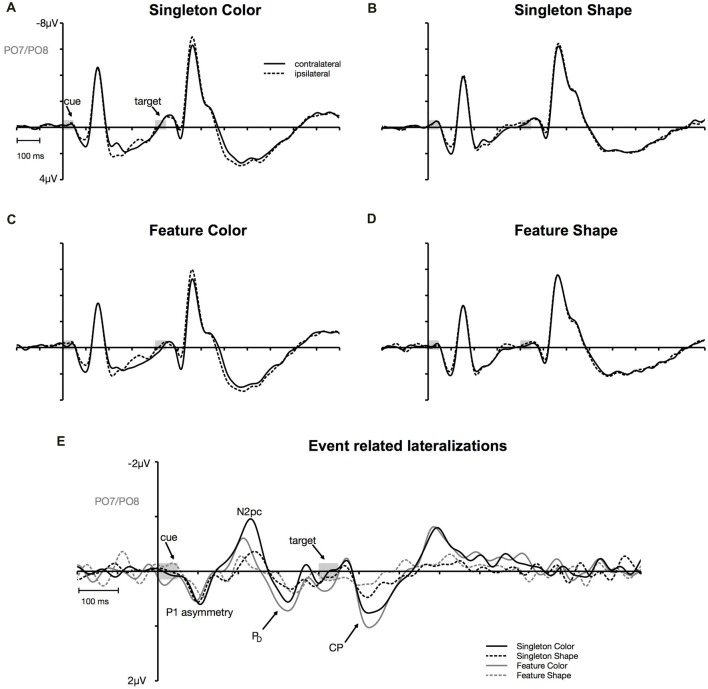
Cue-locked event-related potentials (ERPs) at posterior electrodes PO7/PO8. Contra- and ipsilateral waveforms relatively to the side of the irrelevant color singleton separately depicted for the color and the shape condition of the singleton and feature task **(A–D)**. Contra- minus ipsilateral difference waves (event-related lateralizations, ERLs) time-locked to cue array onset **(E)**.

**Figure 4:**
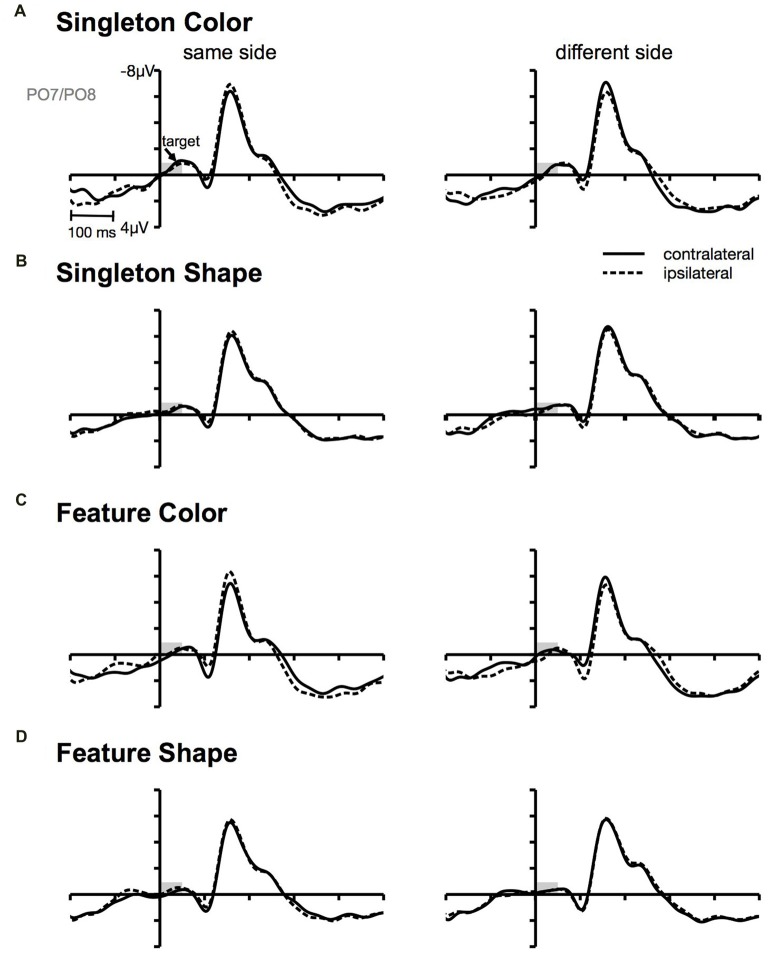
Target-locked ERPs at posterior electrodes PO7/PO8. Contra- and ipsilateral waveforms relatively to the side of the target (same vs. different) separately depicted for the color and the shape condition of the singleton **(A,B)** and feature task **(C,D)**.

We further looked at effects over frontal sites that were strongest at fronto-central electrode FCz. As expected, we observed two distinct *task* effects on frontal components that arose after the onset of the cue: a first effect on the P2 component and a later negative shift in the time window of the N2. Previous findings from spatial cuing studies could also identify modulations of the cue-array-elicited anterior P2 and N2 that differentiated between the amount of top-down control applied during search (Eimer et al., 2009; Verleger et al., 2014). In these investigations, the effect in the time range of the frontal N2 was also not limited to the actual N2 peak. Therefore, the statistical analysis was based on the time window where the negative shift of N2 reached the strongest effect. Mean amplitude measurements were based on the ±20 ms interval around the peak in the grand average. P2 was measured from 154 ms to 194 ms. The negative shift of N2 was assessed by measuring the mean amplitude between 353 ms and 393 ms after cue array onset. We applied an ANOVA with the within-subject factors *task* (singleton, feature) and *target dimension* (color, shape) to test if there were any statistical differences in the components between the different conditions.

For all analyses, *p*-values of <0.05 were considered statistically significant. Adjusted *p*-values (*p*_adj_) are reported when FDR correction was applied to control for multiple comparisons. Partial eta squared ($\eta_{\text{p}}^{2}$) is reported as an indication of effect size.

## Results

### Behavioral Results

RTs and error rates for the two search tasks are separately depicted for the color and the shape condition, as well as for cue and target presented within the same quadrant as the target or at one of the three different locations (see Figure 2). Discrimination errors were generally higher for the shape condition in both the singleton and the feature task, *F*_(1,23)_ = 14.52, $\eta_{\text{p}}^{2}$ = 0.39, *p* < 0.001. Furthermore, we observed more errors in the feature compared to the singleton task *F*_(1,23)_ = 7.17, $\eta_{\text{p}}^{2}$ = 0.24, *p* < 0.05. There was a significant *task* by *target dimension* interaction *F*_(1,23)_ = 5.59, $\eta_{\text{p}}^{2}$ = 0.20, *p* < 0.05: in the shape condition, participants made fewer errors in the singleton than in the feature task, *F*_(1,23)_ = 8.19, $\eta_{\text{p}}^{2}$ = 0.26, *p* < 0.01 whereas there was no differences between the two tasks in the color condition, *F*_(1,23)_ = 1.9, $\eta_{\text{p}}^{2}$ = 0.08, *p* = 0.18. None of the remaining interactions for discrimination errors reached significance, i.e., there were no effects of cue-target location (all *p*-values > 0.13). Participants had overall more omissions in the shape compared to the color task, mirrored by a significant main effect of *target dimension*, *F*_(1,23)_ = 14.14, $\eta_{\text{p}}^{2}$ = 0.38, *p* < 0.01. The analysis revealed a trend toward a significant main effect of cue-target location *F*_(1,23)_ = 3.58, $\eta_{\text{p}}^{2}$ = 0.13, *p* = 0.07. There were no further significant effects in the analysis of omissions (all *p*-values > 0.11).

We found a significant main effect of *target dimension* with faster RTs for the color compared to the shape condition, *F*_(1,23)_ = 278.02, $\eta_{\text{p}}^{2}$ = 0.92, *p* < 0.001. Overall, participants responded more quickly on same compared to different location trials, *F*_(1,23)_ = 14.69, $\eta_{\text{p}}^{2}$ = 0.39, *p* < 0.001. However, this was true in the color condition only, as revealed by the significant *target dimension* by *cue-target location* interaction, *F*_(1,23)_ = 5.93, $\eta_{\text{p}}^{2}$ = 0.20, *p* < 0.05, and effects of cue-target location being significant in the color condition, *F*_(1,23)_ = 13.21, $\eta_{\text{p}}^{2}$ = 0.36, *p* < 0.01, and not in the shape condition, *F*_(1,23)_ = 0.94, $\eta_{\text{p}}^{2}$ = 0.04, *p* = 0.34. No difference in the cuing effect was found between the singleton and the feature task, *F*_(1,23)_ = 0.02, $\eta_{\text{p}}^{2}$ = 0.001, *p* = 0.9. Nor did any of the remaining interactions for RTs reach significance (all *p*-values > 0.1).

### EEG Results

#### Posterior Sites

##### N2pc

**Figure 5:**
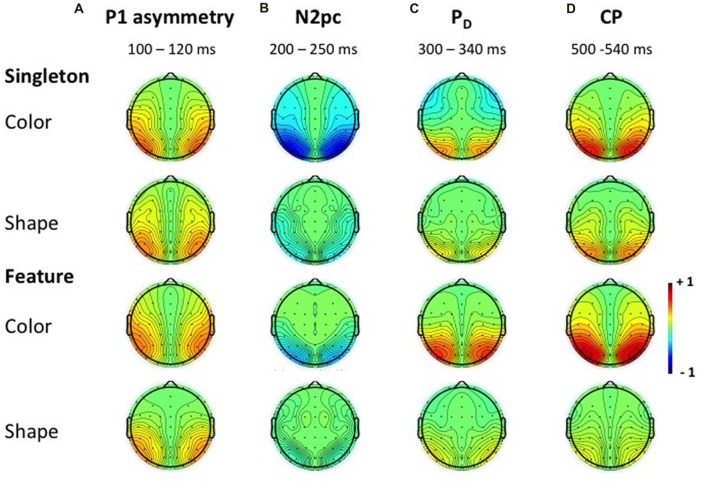
Topographies of the ERLs. Topographies in the time windows of the P1 asymmetry **(A)**, N2pc **(B)**, P_D_ **(C)** and CP **(D)** for the color and shape condition of the singleton and feature task. Because the subtraction was mirrored across both hemispheres, the topographies appear symmetrical.

N2pc appeared as a contralateral negativity that started approximately 180 ms after cue array onset (see Figure 3) and was more pronounced in the color than in the shape condition, *F*_(1,23)_ = 9.35, $\eta_{\text{p}}^{2}$ = 0.29, *p* < 0.01. Furthermore, the effect was generally stronger in the singleton than in the feature task, *F*_(1,23)_ = 7.05, $\eta_{\text{p}}^{2}$ = 0.23, *p* < 0.05. The *task* by *target dimension* interaction did not reach significance, *F*_(1,23)_ = 2.52, $\eta_{\text{p}}^{2}$ = 0.10, *p* = 0.13. The topography in Figure 5 shows that the effect was restricted to posterior sites. An overview of the critical and observed area values of the permutation tests is given in Table 1. Significance was confirmed for the contralateral negativity in the singleton color condition. In the feature color condition, N2pc almost reached significance but remained below the critical level. In the shape conditions of the singleton and feature task, N2pc did not differ from random noise.

**Table 1:** Critical and observed area values of the area permutation tests for the posterior electroencephalogram (EEG) components.

	Singleton		Feature	
	Critical	**Observed**	Critical	**Observed**
**P1**				
Color	*0.015*	0.025*	*0.015*	0.020*
Shape	*0.015*	0.022*	*0.018*	**0.018**
**N2pc**				
Color	*0.030*	0.061*	*0.031*	**0.029**
Shape	*0.031*	**0.019**	*0.036*	**0.010**
**P_D_**				
Color	*0.031*	**0.030**	*0.031*	0.056*
Shape	*0.032*	**0.028**	*0.036*	**0.023**
**CP**				
Color	*0.030*	0.067*	*0.031*	0.079*
Shape	*0.031*	0.037*	*0.034*	**0.016**

*Activation differs from random noise when the observed value exceeds the critical value. The critical value indicates the point where 95% of the values of the random distribution are exceeded. Area values are given in units of μVs. Asterisk indicated significance of the effects*.

##### P_D_

P_D_ started at about 250 ms after cue array onset. ANOVA results revealed a main effect of *target dimension*, *F*_(1,23)_ = 7.26, $\eta_{\text{p}}^{2}$ = 0.24, *p* < 0.05, with overall stronger P_D_ in the contingent color compared to the shape condition. There were no significant differences between the two tasks, *F*_(1,23)_ = 1.16, $\eta_{\text{p}}^{2}$ = 0.05, *p* = 0.29, as well as no *task* by *target dimension* interaction, *F*_(1,23)_ = 1.24, $\eta_{\text{p}}^{2}$ = 0.05, *p* = 0.28. Like N2pc, the effect showed up at posterior sites (see Figure 5). Significant difference from random activation was only confirmed for P_D_ in the color condition of the feature task (see Table 1). In both, the color and shape condition of the singleton task, P_D_ slightly missed significance. No significant difference from random noise could be confirmed for P_D_ in the shape condition of the feature task.

##### CP

We furthermore observed a second contralateral positivity after target array onset (see Figure 3). This CP started at approximately 480 ms and was restricted to posterior sites (see Figure 5). Statistical analyses revealed a significant *task* by *target dimension* interaction, *F*_(1,23)_ = 5.05, $\eta_{\text{p}}^{2}$ = 0.18, *p* < 0.05. The difference in activation of CP between the color and the shape condition was stronger for the feature, *F*_(1,23)_ = 30.75, $\eta_{\text{p}}^{2}$ = 0.57, *p*_adj_ < 0.001, than for the singleton task, *F*_(1,23)_ = 11.8, $\eta_{\text{p}}^{2}$ = 0.48, *p*_adj_ = 0.005 (see Figure 3). For the color condition, the difference between the tasks did not reach significance *F*_(1,23)_ = 1.56, $\eta_{\text{p}}^{2}$ = 0.06, *p*_adj_ = 0.22. When comparing the strength of CP between the shape condition of the singleton and the shape condition of the feature task, statistical analysis revealed a significant effect for this difference, *F*_(1,23)_ = 6.39, $\eta_{\text{p}}^{2}$ = 0.22, *p*_adj_ = 0.03. Permutation tests confirmed a significant difference from random noise in the time window of CP for the color conditions of the singleton and feature task as well as for the shape condition of the singleton task (see Table 1). For the feature shape condition, however, the effect was not reliably present.

The analysis for the P1 asymmetry revealed no significant differences between the conditions (all *p*-values > 0.3). Permutation analyses verified that the effect in the time window of the P1 significantly differed from zero except for the feature shape condition (see Table 1).

#### Frontal Sites

**Figure 6:**
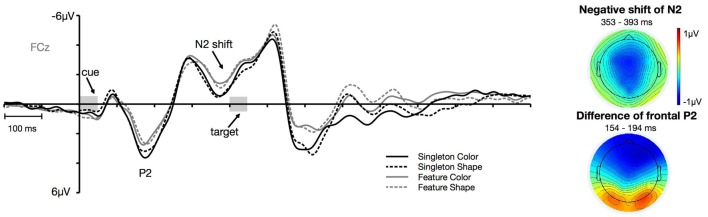
Frontal ERPs at electrode FCz. The frontal P2 effect and the negative shift of the frontal N2 separately depicted for the color and shape condition of the singleton and feature task. The topographies depict the difference between the feature and singleton task in the time window of the P2 and the negative shift of the N2 component.

Figure 6 shows the cue-locked ERPs at frontal electrode FCz for the color and shape condition in the singleton and feature task. First of all, there arose a positivity at about 140 ms after cue array onset. This P2 was generally more pronounced for the singleton task, *F*_(1,23)_ = 9.98, $\eta_{\text{p}}^{2}$ = 0.30, *p* < 0.01, with no differences between the color and the shape condition, *F*_(1,23)_ = 1.15, $\eta_{\text{p}}^{2}$ = 0.05, *p* = 0.3. Furthermore, we observed a negative shift that started in the time window of the N2 (at about 340 ms after the onset of the cue array). This negativity was overall stronger for the feature compared to the singleton task, *F*_(1,23)_ = 8.15, $\eta_{\text{p}}^{2}$ = 0.26, *p* < 0.01. Like for the P2, there was no difference in mean amplitude between the color and shape conditions, *F*_(1,23)_ = 0.14, $\eta_{\text{p}}^{2}$ = 0.01, *p* = 0.72. None of the *task* by *target dimension* interactions reached significance, *F*_(1,23)_ = 2.73, $\eta_{\text{p}}^{2}$ = 0.11, *p* = 0.11 (for P2), and *F*_(1,23)_ = 0.27, $\eta_{\text{p}}^{2}$ = 0.01, *p* = 0.61 (for N2).

## Discussion

An attentional template may be regarded as the helmsman that brings our focus of attention on the desired course. This top-down bias is of central importance for our behavior as it highlights currently relevant information and thereby promotes its selection (Bundesen, 1990; Duncan and Humphreys, 1992; Desimone and Duncan, 1995; Chelazzi et al., 1998). By means of a spatial cuing paradigm, we aimed to investigate how cognitive control is altered with the scope of an attentional template currently activated in visual working memory. Participants had to search for a target item that was preceded by a display containing an irrelevant red singleton cue. By implementing four different search arrays we tried to trigger different manifestations of attentional templates in order to find out if there emerge any differences in the amount of cognitive control during search. Blockwise, the target was either a red circle or a gray square, but had to be searched within homogenous (gray circles) or heterogeneous non-targets (differently colored circles or various shapes). Thus, in two conditions the target was a singleton and could be either detected by adopting a broader, salience-based or a narrower, feature-specific template. For those conditions where the target was defined by a specific feature, a more fine-tuned template had to be activated in working memory. We expected the amount of cognitive control to be overall enhanced when search is accomplished on the basis of a feature-specific template.

Behavioral results showed that the singleton cue in the first display captured attention whenever it matched the target in the specific feature dimension, thus when participants had to search for the red circle (singleton and feature color condition). In the shape condition, behavioral spatial cuing effects failed to appear on RT level regardless of whether the shape was defined as a singleton or was presented within different shapes. It should be mentioned that for omissions, participants tended to make overall fewer errors on same compared to different location trials. This indicates that the singleton cue summoned attention to its location regardless of whether it was contingent on the attentional set or not. Thus, some sort of salience-based capture was triggered by the pop-out stimuli in the cue array.

Whereas the behavioral findings did not allow for a differentiation between the amount of cognitive control applied during the singleton and feature task, the EEG data provided evidence that different scopes of templates were established dependent on the task at hand. At posterior sites, this difference in the amount of top-down control became apparent in modulations of N2pc. Selection of the irrelevant information indexed by N2pc featured a stronger effect in the color compared to the shape condition and in the singleton compared to the feature task. This suggests that participants applied more control over the orienting of attention to irrelevant information, as a consequence of a feature-specific template compared to a template based on the search for a pop-out element in the display.

P_D_ arose after N2pc and before target array onset. This contralateral positivity referred to the location of the irrelevant cue indexes the neutralization of the selected information (Hickey et al., 2009; Sawaki and Luck, 2013) but was shown to be independent from the actual strength of capture indexed by N2pc (Mertes et al., 2016, 2017). In line with this assumption, P_D_ displayed no difference between the singleton and the feature task, but was stronger for the color compared to the shape condition. This inhibitory mechanism reflected by P_D_ was presumably engaged to re-orient attention to the central position in order to adequately process the target array.

Furthermore, we observed a contralateral positivity referred to the location of the irrelevant cue that was elicited by the target array (i.e., CP). From Figure 4, it can be seen that the CP originated from a contralateral relative to ipsilateral P1 enhancement of the stimulus in the search array that appeared at the same location as the preceding cue. There exists some ambiguity regarding the functional meaning of this target-array-elicited effect in a spatial cuing paradigm. Long-standing evidence suggests that P1 asymmetry might reflect a boost in perceptual processing of the cued stimulus in the target array (Heinze et al., 1990; Luck et al., 1990; McDonald et al., 2005; Fukuda and Vogel, 2009; Störmer et al., 2009; Livingstone et al., 2017). Störmer et al. (2009) could for example show that in the presence of more pronounced CPs, participants reported a higher contrast of the cued target. Thus, guiding attention to one of two identical targets led to an increase of the perceived stimulus contrast by enhancing early sensory mechanisms. Similarly, Fukuda and Vogel (2009) observed an enhanced contralateral P1 that was followed by an ipsilateral enhancement of N1 for stimuli presented at attended locations. This finding resembles the P1/N1 modulation observed in the current investigation with a stronger CP effect in the color compared to the non-contingent shape task (see Figure 3). Referred to the cuing effect observed on behavioral level, this attentional enhancement at early perceptual stages facilitated the processing of the target on same side trials, but led to interference on different side trials. This could account for the RT difference between same and different location trials in the color condition of the singleton and feature task (Livingstone et al., 2017).

Nonetheless, it is impossible to interpret the current findings regarding the CP exclusively in the light of attentional modulation on the sensory processing of the target array. This account postulates that the orienting of attention toward the irrelevant cue singleton leads to a spatially specific effect regarding the sensory processing of the target array. The CP effect should, therefore, be related to the extent of the posterior asymmetries following the cue display. To be more specific, CP should be largest when correlates of attentional capture are most pronounced (i.e., N2pc). We did, however, not observe this pattern of results in the current experiment. While N2pc was larger in the singleton compared to the feature task, we did not observe this main effect for the CP. The difference between the CP effect in the color vs. shape condition was larger for the feature search template compared to the singleton template. To be more specific, there was no evidence for a CP effect in the feature shape condition, but a strong effect in the feature color condition. In the singleton condition both CP effects were significant. We propose that these variations in the CP effect are in part related to an inhibitory control process during target processing: inhibition might have been engaged to compensate the detrimental influence of spatial cue information, with the strength of this process varying with the attentional template activated in WM. Regarding the spatial cuing effect on behavioral level, this would imply that the CP reflects the compensation of the former attentional distraction. The need for such a mechanism should be higher in the contingent compared to the non-contingent condition.

Additionally, the differences in CP amplitude based on the attentional template that was adopted during search might be explained in the light of this inhibition account. When more top-down control was engaged by means of the feature-specific attentional template, the following cascade of attentional orienting might have been triggered in the color condition: capture by the irrelevant information was reduced (N2pc) and further counteracted during target processing (CP). However, the absence of any posterior asymmetries in the shape condition of the feature task might suggest that the adoption of a specific template for the search of a target in a heterogeneous display was sufficient to prevent attentional orienting to non-contingent irrelevant cues. The search template might have been so precise that it only included the specific attributes of the target (i.e., red circle or gray square), thereby reducing attentional capture by the irrelevant cue.

A further indication in support of the inhibition account can be derived from previous studies. Mertes et al. (2016) could only find a contralateral enhancement of the target-evoked P1 for those trials where cue and target were presented at the same side. When the target appeared at the different side referred to the preceding cue, the effect shifted in latency and was only evident as a contralateral reduction of N1. It is not possible to explain this finding in the light of a boost of early sensory mechanisms. In an aging study conducted by Mertes et al. (2017), it was shown that in the presence of a reliable N2pc effect and attentional capture at the behavioral level, older adults lacked the target-array-elicited positivity in a spatial cuing paradigm. As older adults suffered from stickiness of visual processing (pronounced cuing effects for older compared to younger adults), it was supposed that inhibitory mechanisms contributed to the late contralateral positivity.

In addition, we observed a contralateral asymmetry in the time window of the cue-related P1 that was present in all conditions except the feature shape condition where a significant effect was only slightly missed. This early contralateral positivity somewhat resembles the so-called Ppc (Leblanc et al., 2008; Fortier-Gauthier et al., 2012; Jannati et al., 2013). Actually, the Ppc had a slightly later onset than the contralateral P1 in the present study as it usually overlapped with the time window of the N1. Nevertheless, this component has been triggered by target and non-target singletons particularly during fixed feature search and has been observed over lateral occipital electrode sites. Therefore, the Ppc shares some characteristics with the contralateral positivity that was elicited by the cue array in the current investigation. This early asymmetry might be associated with laterally imbalanced sensory activity (Luck and Hillyard, 1994a; Kimura et al., 2008). As we controlled for luminance, we would, however, exclude the possibility that the P1 asymmetry was caused by perceptually imbalanced stimulus presentation. An additional indication that this early contralateral positivity did not index purely sensory-driven processes was revealed by an investigation of Fortier-Gauthier et al. (2012), showing that the Ppc was triggered by a centrally presented probe. Furthermore, the Ppc might be an early index of distractor suppression (Sawaki and Luck, 2010). The fact that older adults lack the contralateral enhancement of P1 while suffering from a reduced ability to inhibit the irrelevant cue during contingent attentional orienting (see Mertes et al., 2017) supports the inhibitory function of this component. In the current context, however, the contralateral P1 effect neither helped to explain the different development of the subsequent components nor could it account for the behavioral effects that differentiated between the color and the shape condition. In line with our observation, Jannati et al. (2013) found a Ppc in response to singletons regardless of whether they were relevant or irrelevant for the task at hand. This early asymmetry had also no effect on target processing and might probably index salience-based selection of the red singleton rather than any attention-related or inhibitory process. Further research is needed to elucidate the specific conditions triggering the occurrence of this contralateral P1.

We might wonder why the template-based differences in top-down control observed in the current study were not reflected by modulations in spatial cuing effects. Such a dissociation between behavioral and electrophysiological findings was already reported by previous investigations (Hopfinger and Ries, 2005; Eimer and Kiss, 2008; Grubert and Eimer, 2016; Mertes et al., 2016, 2017). For example, Eimer and Kiss (2010) reported reduced but reliable N2pc components to irrelevant cues in the absence of behavioral cuing effects when participants searched on the basis of a feature-specific template. Thus, in some cases the irrelevant cue summoned attention to its location implying that it was part of the currently activated attentional template. Either on an inter- or intra-individual level (or even both), participants might have switched between singleton and feature templates in this condition. Based on the assumption that there is always the attempt to reduce cognitive effort during search (Bacon and Egeth, 1994), the adoption of an easily implemented singleton template would have made perfect sense in the singleton task of the current study. Leber and Egeth (2006) even argued that a drop in search performance due to the adoption of a singleton template might be accepted up to a certain point as a trade-off with cognitive effort. But there is also good reason for the adoption of a feature-specific template in search for the shape singleton: in the current design, the net cost between using a singleton or a feature template should be the same, because in both cases the template includes only one element (looking for the square vs. looking for a singleton). As the target and distractor features were held constant across all trials within the respective blocks, the adoption of a shape template might have been the more efficient choice as it prevents distraction by the irrelevant cue (Grubert and Eimer, 2016). Thus, singleton detection mode does not seem to be the default strategy in any case. On the basis of the requirements of the task and the characteristics of the stimuli, participants rather voluntarily select the strategy that reduced attentional costs on an individual level. This possible switch in the scope of the attentional template might account for the differences in the correlates of attentional capture between the behavioral and electrophysiological level observed in the current investigation. In addition, Hopfinger and Ries (2005) showed that also target-related effects in the EEG might be dissociated from behavioral outcomes. A target-evoked contralateral P1 enhancement was evident when cue and target were presented at the same location regardless of whether the cue was contingent on the attention set or not. Only the latency of this effect varied with the currently adopted top-down control set. Behavioral spatial cuing effects showed up as expected, namely only in the contingent condition. The authors concluded that early bottom-up effects might be compensated by later top-down mechanisms leading to different behavioral outcomes.

Further support for the assumption that cognitive control was enhanced in the feature compared to the singleton task was provided by ERP effects at frontal scalp sites. Our analyses revealed two components that differentiate between these experimental conditions. First of all, we observed a P2 that was overall pronounced for the singleton compared to the feature task. In a spatial cuing paradigm, Verleger et al. (2014) reported a similar effect: They found an enhanced positivity for color cues that were contingent on the currently activated template in working memory. Verleger et al. (2014) stated that this effect might reflect activation induced by the cue when it matched the target properties. In our study, we did not find any differences between contingent and non-contingent conditions. The frontal effect rather varied with the amount of cognitive control engaged due to the adoption of different attentional templates. Thus, the enhanced positivity in the singleton task might reflect a deeper processing of the color cue when cognitive control is reduced due to a more saliency-based attentional orienting (i.e., singleton template). This assumption follows the interpretation of Verleger et al. (2014) and is furthermore in line with other considerations regarding the meaning of frontal P2 as a selection positivity (Kenemans et al., 1993; Anllo-Vento and Hillyard, 1996) or an index of attentive and feature-based stimulus evaluation (Potts, 2004). In addition, we observed a negative shift starting after the N2 peak that was generally enhanced for the feature task. This effect is reminiscent of the anterior N2 reported in a study of Eimer et al. (2009). In a spatial cuing paradigm, irrelevant cues that were non-contingent on the attentional set evoked a more pronounced frontal N2 than did contingent cues. Even if this effect occurred earlier then the frontal shift in the present investigation, it also outlived the actual N2 peak. Eimer et al. (2009) supposed that this anterior N2 indexed a mechanism of inhibitory control that was engaged to prevent attentional capture by the irrelevant information that did not match the currently activated search template. This is in line with other interpretations of the frontal N2 as a mechanism of cognitive control during the processing of irrelevant information (Falkenstein et al., 2002; Folstein and Van Petten, 2008) or conflict processing (van Veen and Carter, 2002). In addition, also the study of Verleger et al. (2014) revealed higher negativity in the N2 time window for non-contingent than for contingent irrelevant singletons. The shape such as the time course of this effect is largely comparable to the negative shift of N2 observed in the present study. Similar to the frontal P2, we could only observe a global differentiation of the N2 effect between the singleton and the feature task. Therefore, we would conclude that the enhanced negative shift in the feature task is a further hint for the adoption of a stricter template with a narrower attentional scope that resulted in enhanced top-down control of the irrelevant cue.

There are some limitations of the present study that must be mentioned: First of all, we did not balance the cue type according to the different target conditions as it was always defined as a red singleton. Pomerleau et al. (2014) found evidence that attention is more efficiently deployed to red stimuli, even when they are presented equiluminantly. Therefore, it could be suspected that choosing red singletons might have an additional influence on the allocation of attention and added to the effect of contingent attentional orienting. This might also account for the occurrences of the early asymmetry in the time window of the P1 in the present investigation as Pomerleau et al. (2014) could show an enhancement of the Ppc when the target was presented in red. In contrast, Mertes et al. (2016) could not find any differences in the early contralateral positivity regardless of whether the irrelevant cue was always presented in red or appeared randomly in either red, green, yellow or blue.

Furthermore, we implemented a slightly longer SOA (i.e., 400 ms) than did previous studies, which commonly used an offset of 150 or 200 ms between the cue and the target array (e.g., Eimer and Kiss, 2008; Eimer et al., 2009). Therefore, the attention-grabbing influence of the cue might have been reduced due to additional time available to re-orient the attentional focus. This could be another reason for the dissociation between behavioral and electrophysiological data in the course of the current study. In addition, shape targets produced essentially longer RTs than color targets which might have weakened the cuing effect in these conditions.

Altogether, the ERP findings at posterior and frontal sites are in line with our assumption of stronger cognitive control of irrelevant information when a more fine-tuned feature template is adopted: Reduced selection mirrored by N2pc and P2 and enhanced inhibition reflected by the negative shift of N2. The fact that this distinction could not be made based on the behavioral findings points toward a more fine-grained and stepwise implementation of top-down control during visual search.

## Author Contributions

DS and CM conceived the experimental design and performed the interpretation, revision and final editing of the work. CM carried out the experiment, collected the data, conducted the analysis of the behavioral and electrophysiological data and wrote the manuscript.

## Conflict of Interest Statement

The authors declare that the research was conducted in the absence of any commercial or financial relationships that could be construed as a potential conflict of interest.
